# Agricultural input shocks affect crop yields more in the high-yielding areas of the world

**DOI:** 10.1038/s43016-023-00873-z

**Published:** 2023-11-09

**Authors:** Aino Ahvo, Matias Heino, Vilma Sandström, Daniel Chrisendo, Mika Jalava, Matti Kummu

**Affiliations:** Water and Development Research Group, Aalto University, Espoo, Finland

**Keywords:** Sustainability, Agriculture

## Abstract

The industrialization of agriculture has led to an increasing dependence on non-locally sourced agricultural inputs. Hence, shocks in the availability of agricultural inputs can be devastating to food crop production. There is also a pressure to decrease the use of synthetic fertilizers and pesticides in many areas. However, the combined impact of the agricultural input shocks on crop yields has not yet been systematically assessed globally. Here we modelled the effects of agricultural input shocks using a random forest machine learning algorithm. We show that shocks in fertilizers cause the most drastic yield losses. Under the scenario of 50% shock in all studied agricultural inputs, global maize production could decrease up to 26%, and global wheat production up to 21%, impacting particularly the high-yielding ‘breadbasket’ areas of the world. Our study provides insights into global food system resilience and can be useful for preparing for potential future shocks or agricultural input availability decreases at local and global scales.

## Abstract (web summary)

The relationships between the use of agricultural inputs and crop yields are complex. This study presents a machine learning model to predict the changes in crop yield in the face of single and combined agricultural input shocks and shows that shocks in the availability of commercial agricultural inputs have a considerable impact on crop yields, particularly in high-yield regions worldwide.

## Main

The industrialized food production systems, on which the majority of global food crops are grown^1^, depend on off-farm inputs such as synthetic fertilizers, machinery, energy, pesticides, seeds and animal feed. Many of these inputs are imported, often only from a few countries^2^. And while some countries might be resilient enough in their food system to handle local extreme weather shocks or disturbances in food trade flows, their food production might be influenced by the availability of agricultural inputs^3,4^. Also, there is an increasing pressure to decrease the use of synthetic fertilizers and pesticides to reduce their burden on environment^5,6^.

The relationships of different disturbances on food production (extreme weather and so on) and supply (trade shocks and so on) are increasingly well understood (for example, Dall’erba et al.^7^ and Ferguson and Gars^8^). Very little is known, however, how drastically decreased use or availability of agricultural inputs would impact food crop yields and thus food availability and food security on a large scale (national or global). The few existing studies include, for example, Beckman et al.^5^ who, using economic models, study the effects of globally adopting the European Union’s (EU’s) Green Deal and Farm to Fork strategies that aim to reduce pesticides by 50% and fertilizers by 20%. Responses to input reductions differed greatly around the globe, but, for example in the EU, wheat production would decrease by 33%. Jansik et al.^9^, in turn, use expert interviews to investigate the effects of agricultural input shocks on Finnish agriculture. In their estimate, a total shock in the input of farm chemicals, fertilizers and pesticides could reduce yields by 10–40% (crops not specified).

In this Article, we aim to assess the impacts of combined agricultural input shocks (or decrease by other reasons) on crop yields globally with 5 arcmin resolution (10 km at the equator) and map the areas and crops that are particularly vulnerable to the shocks or decreased input use levels. We also identify the input shocks that would most decrease the yield separately for each crop type. Characterizing high-risk areas and crops will provide crucial information on national and global food security in the times of global geopolitical turmoil.

**Extended Data Fig. 1:**
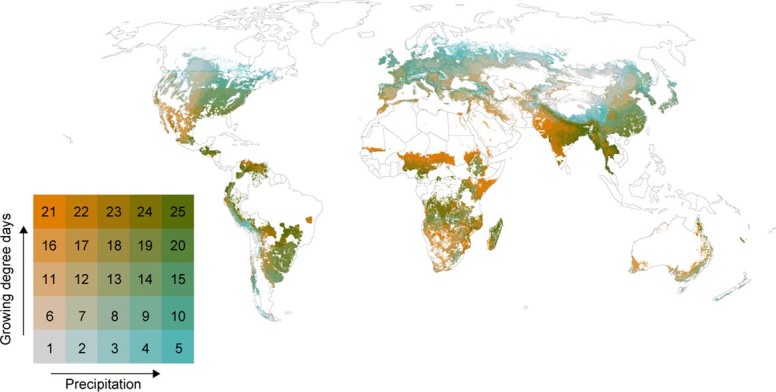
Climate bins for wheat. Each climate bin represents an area with similar Growing Degree Day (GDD) and precipitation characteristics and has roughly similar amount of grid cells. Spatial resolution is 5 arc-min. The GDD and precipitation thresholds are shown in Extended Data Fig. 2. See climate bins for all crops at Agri.Input.Shock -explorer: http://193.166.24.46:3838/shock_shiny/.

**Extended Data Fig. 2:**
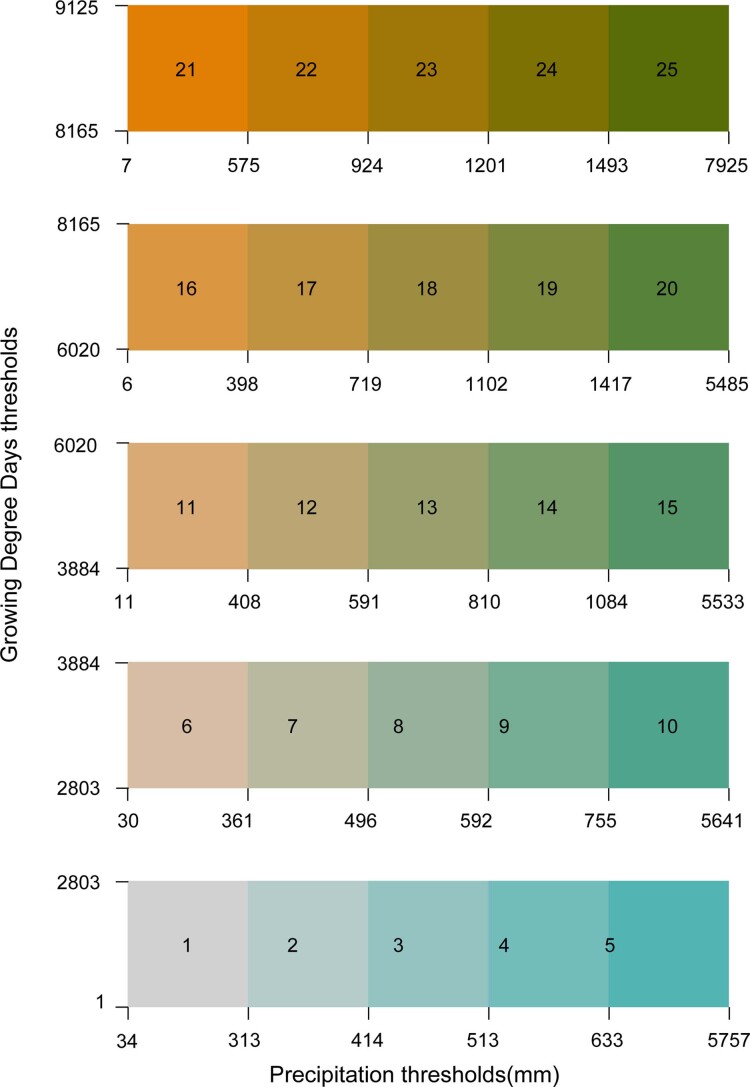
Growing degree day and precipitation thresholds for the climate bins of wheat. See map of the climate bins in Extended Data Fig. 1.

We used a random forest machine learning model^10^ to conduct the assessment. The model allowed us to estimate the impact of different levels of agricultural input shocks (25%, 50% and 75%) as well as different shock combinations (nitrogen (N), phosphorus (P) and potassium (K) fertilizer shocks individually, machinery shock, pesticide shock, shock in all fertilizers together and shock in all inputs together) on agricultural yields of 12 food crops globally (Methods). We controlled the impact of climate by dividing the globe to 25 climate bins (see example map of climate bins in Extended Data Fig. 1 and thresholds for growing degree days (GDD) and precipitation in Extended Data Fig. 2). In addition, we considered soil conditions by adding three soil parameters to the model (Methods). Using spatially gridded datasets allowed us to model the effects in high resolution and identify sub-national differences that are hindered in national-scale analyses. Our results reveal the areas where decrease in agricultural input use would have the highest hit on yields and thus potentially threatening food security and, inversely, where the yields would not necessarily be that hardly impacted.

## Results

**Extended Data Fig. 3:**
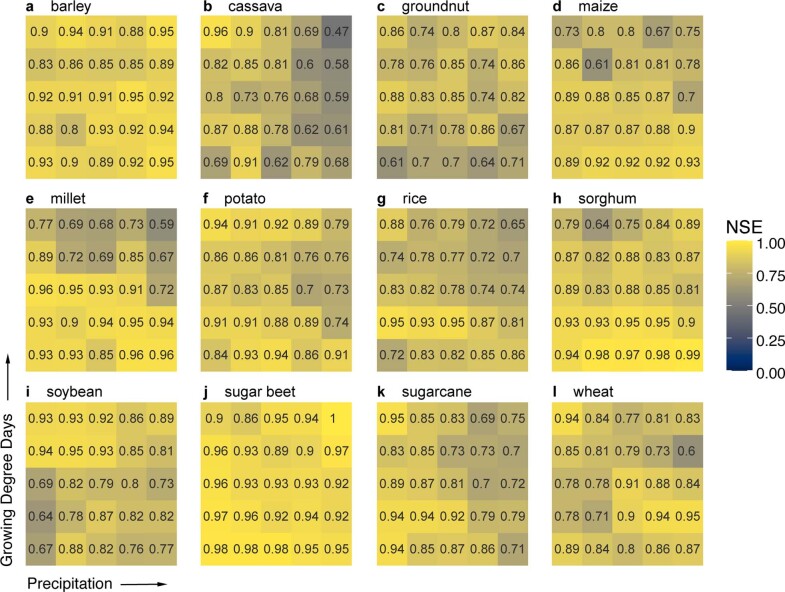
Model performance at grid cell level across climate bins. Results are shown for each crop separately: a. barley, b. cassava, c. groundnut, d. maize, e. millet, f. potato, g. rice, h. sorghum, i. soybean, j. sugar beet, k. sugarcane, and l. wheat. Model performance estimated by comparing the model predictions of the testing data to the known original yields using Nash-Sutcliffe model efficiency (NSE). Scores are shown for all crops and climate bins (n = 25). A score above 0.5 denotes a satisfactory model, above 0.65 a good model and above 0.75 a very good model 21. See climate bin for wheat, as an example, in Extended Data Fig. 1.

**Extended Data Fig. 4:**
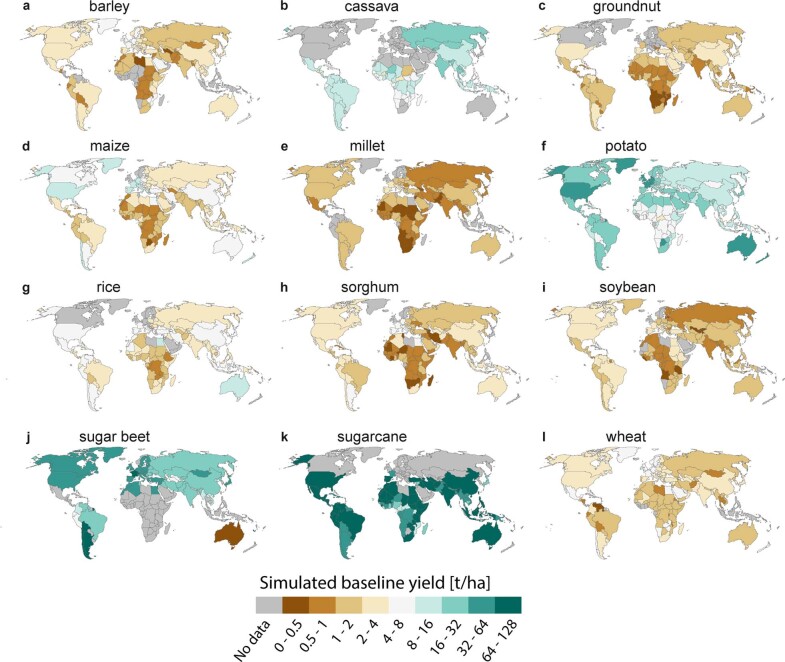
Average country level modelled baseline crop yield (t/ha) for the 12 crops included in the study. Results are shown for each crop separately: a. barley, b. cassava, c. groundnut, d. maize, e. millet, f. potato, g. rice, h. sorghum, i. soybean, j. sugar beet, k. sugarcane, and l. wheat.

**Extended Data Fig. 5:**
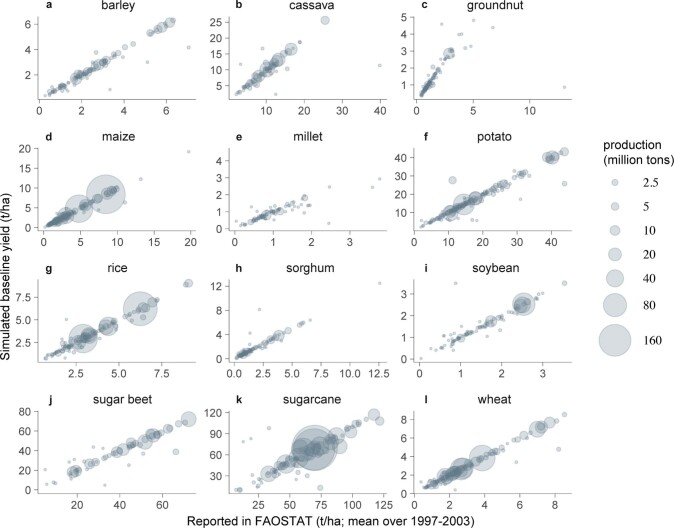
Model performance at country level. Results are shown for each crop separately: a. barley, b. cassava, c. groundnut, d. maize, e. millet, f. potato, g. rice, h. sorghum, i. soybean, j. sugar beet, k. sugarcane, and l. wheat. Comparison of the modelled mean country level yields (y-axis) with the reported yields from FAOSTAT 11 (x-axis), averaged over years 1997-2013. Bubble size represents the country level modelled production of the crop. See summary statistics in Supplementary Table 1.

**Extended Data Fig. 6:**
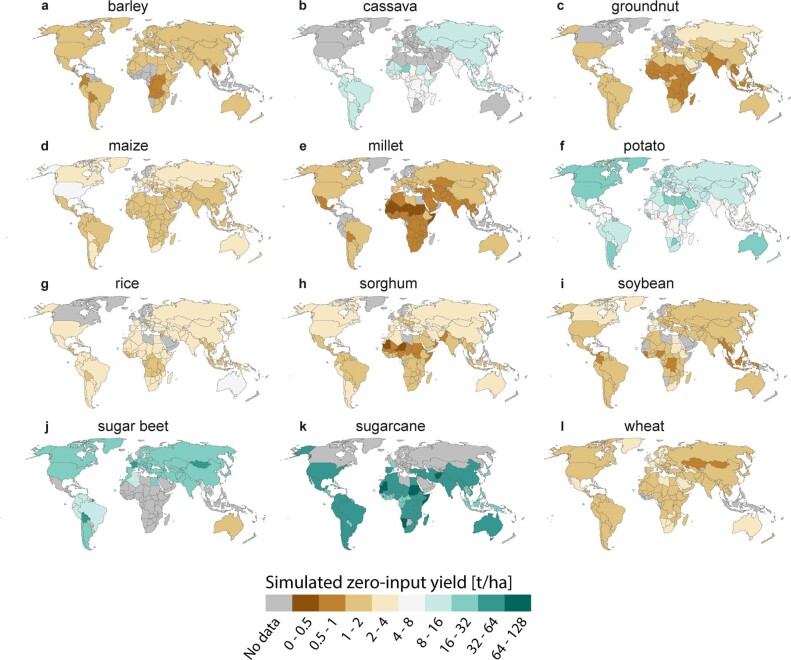
Average country level modelled crop yield (t/ha) with zero agricultural input for the 12 crops included in the study. Results are shown for each crop separately: a. barley, b. cassava, c. groundnut, d. maize, e. millet, f. potato, g. rice, h. sorghum, i. soybean, j. sugar beet, k. sugarcane, and l. wheat.

**Extended Data Fig. 7:**
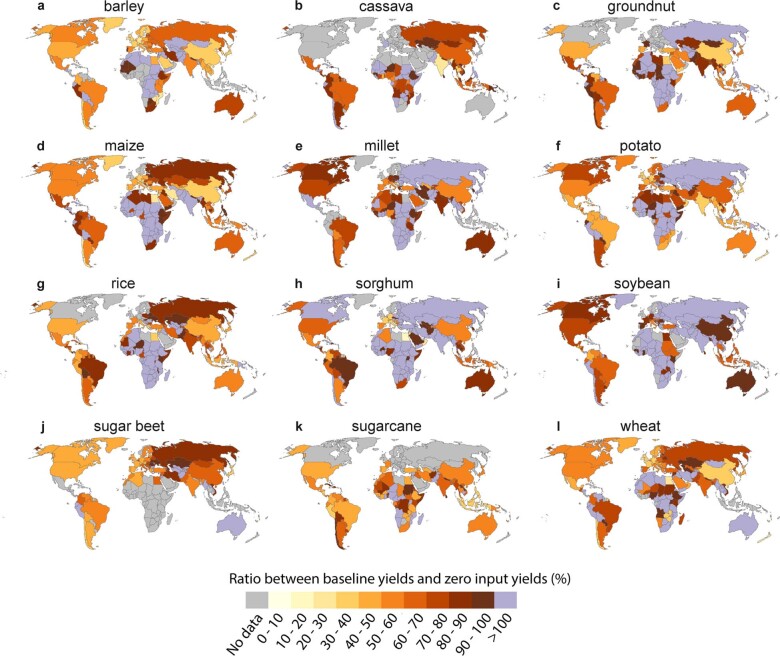
Ratio between crop yield from the modelled scenario with zero agricultural input and the modelled baseline scenario. Results are shown for each crop separately: a. barley, b. cassava, c. groundnut, d. maize, e. millet, f. potato, g. rice, h. sorghum, i. soybean, j. sugar beet, k. sugarcane, and l. wheat. The smaller the ratio, the more the yields decreased in zero input scenario compared to the baseline. In case of ration over 100%, the yields increased (see Discussion in the main text).

Before applying the developed random forest model to input shock scenarios, we estimated the model performance by comparing the model predictions of the testing data to the known original yields separately for each climate bin and crop (see Methods). For most of the crop - climate bin combinations the model performance was good (Nash–Sutcliffe efficiency (NSE) 0.65–0.75; 16% of the models) or very good (NSE > 0.75; 79% of the models) (Extended Data Fig. 3). We also validated our simulated baseline yields (Extended Data Fig. 4) against Food and Agriculture Organization of the United Nations Statistics (FAOSTAT) reported yields^11^ with very good agreement (*R*^2^ > 0.85 for all crops when weighted with production of each country; Supplementary Table 1 and Extended Data Fig. 5). In addition, the validation results from a control scenario (where all inputs were set to zero) show an appreciable yield and thus further confirm the robustness of the model (Extended Data Figs. 6 and 7). More visualizations of model performances and behaviours can be viewed in the Agri.Input.Shock -explorer (http://193.166.24.46:3838/shock_shiny/).

### Agricultural input shocks affect yields

The validated model allowed us to estimate the impact of different kinds and levels of agricultural input shocks on yields for each crop–climate bin combination. The model estimates the change in yield of a shock scenario for a given grid cell by screening areas within a climate bin (where this grid cell is located) and where input use in the baseline is similar to the scenario input use of the target grid cell. The decreased yields, after the shocks were applied, indicate that within the climate bin in question, the baseline yields were only attainable with original input values. Increased yields after scenario shocks mean that in the same climate bin, similar or better yields are possible with less commercial agricultural inputs.

**Fig. 1:**
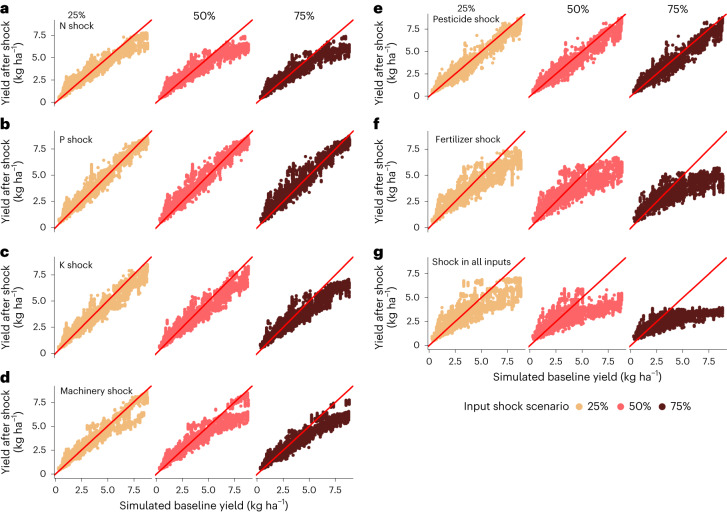
Impacts of input shocks on wheat yield in a single climate bin. Relationship between modelled baseline and scenario yields for each of the studied shock scenarios for one crop–climate bin combination (wheat bin 10).**a**–**g**, Results for each shock scenario: N (nitrogen) shock (**a**); P (phosphorous) shock (**b**); K (potassium) shock (**c**); machinery shock (**d**); pesticide shock (**e**); fertilizer shock (**f**); shock in all inputs (**g**). The red diagonal line denotes the 1:1 line where scenario yields would be identical to baseline yields. Points below the red line indicate that the agricultural input shock decreased the yields. See plots for all crop–climate bin combinations in Agri.Input.Shock -explorer: http://193.166.24.46:3838/shock_shiny/.

As an example of all the applied shock scenarios, the results for wheat in climate bin 10 are shown in Fig. 1—being representant of overall impacts of shocks on different crops–climate bin combinations (see all results in Agri.Input.Shock -explorer). The areas with the highest original yields suffered the most when subjected to the shocks, as was the case also for most scenarios in all crops and climate bins. If the original yield was smaller, the shock yield stayed the same or may have even increased. As expected, the larger input shock scenarios decrease the yields more: 75% input shock resulted in the lowest shock yields.

For example, for wheat in climate bin 10, shocks in the P rate and pesticide do not show large declines in yield, while yields are decreased by shocks in the N rate, K rate and machinery as well as a combined fertilizer shock and a shock in all inputs (Fig. 1). Similar responses are seen in all crops, with the scenarios of largest impact somewhat varying between crops and climate bins (Agri.Input.Shock -explorer).

**Fig. 2:**
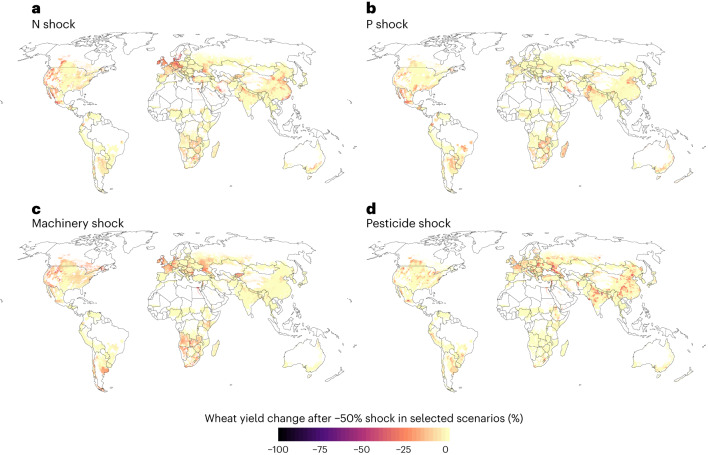
Wheat yield change after a 50% shock in selected inputs. **a**–**d**, Results for each input shock scenario: N (nitrogen) shock (**a**); P (phosphorous) shock (**b**); machinery shock (**c**); pesticide shock (**d**). Note: here only the decreases in yields are shown, and the agricultural areas where yield might potentially increase following a shock are shown in Extended Data Fig. 8. See all shock scenarios for all crops in Agri.Input.Shock -explorer: http://193.166.24.46:3838/shock_shiny/.

**Fig. 3:**
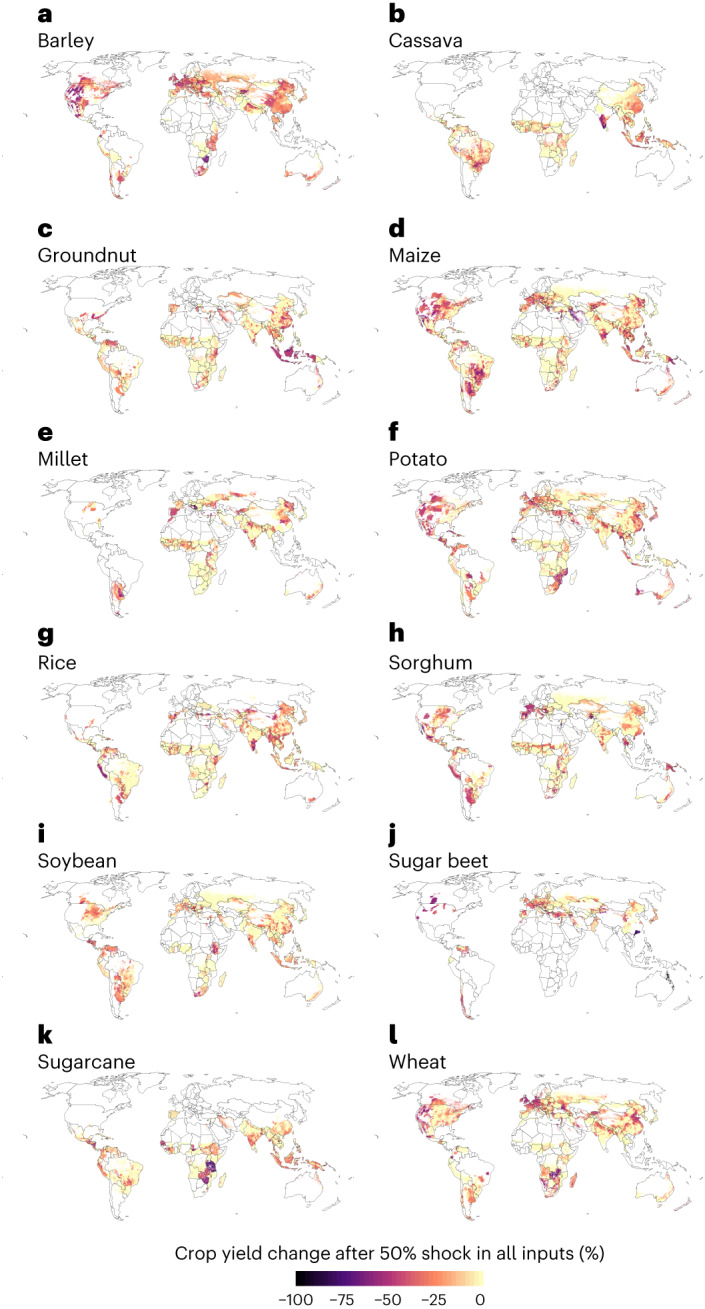
Crop yield change across all crops after a 50% shock in all inputs. **a**–**l**, Results for each crop: barley (**a**); cassava (**b**); groundnut (**c**); maize (**d**); millet (**e**); potato (**f**); rice (**g**); sorghum (**h**); soybean (**i**); sugar beet (**j**); sugarcane (**k**); wheat (**l**). Note: here only the decreases in yields are shown, and the agricultural areas where yield might potentially increase following a shock are shown in Extended Data Fig. 9. See all shock scenarios in Agri.Input.Shock -explorer: http://193.166.24.46:3838/shock_shiny/.

When exploring the impacts of the scenario with 50% shock in N input geographically, we found that wheat yield decreases were particularly induced in, for example, Central Europe, parts of North America and some locations in Southern Africa, China and India (Fig. 2a). However, a combined shock in all inputs decreased wheat yields more—and in more locations (Fig. 3l)—than only N-input shock. Crops were impacted differently by the input shocks. When analysing the combined shock in all inputs, some areas showed decreased yields for multiple crops, but there were no areas with declined yields for all crops (Fig. 3). The yields of barley, maize, potato and wheat all decreased heavily in the western part of the United States. Barley, maize, millet, potato, sorghum and soybean yields all decreased in the northern part of Argentina, while barley, maize, potato, wheat and to some extent sugar beet also saw large yield decreases in Central Europe (particularly in France, Germany and the UK). Rice yields in turn decreased heavily in Thailand, Vietnam and the southern part of India (Fig. 3g).

**Extended Data Fig. 8:**
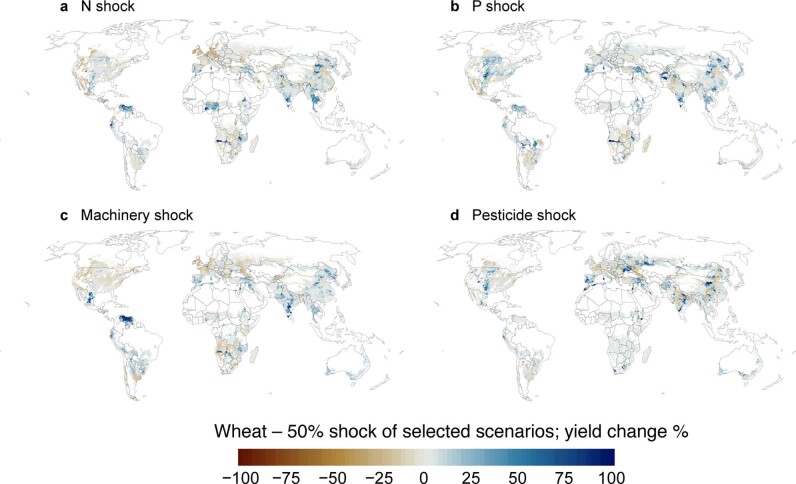
Wheat yield change after a 50% shock in selected inputs. Results are shown for each input shock scenario separately: a. N (nitrogen) shock, b. P (phosphorous) shock, c. machinery shock, and d. pesticide shock. Here both decreased and increased yields are shown, otherwise the figure is similar to Fig. 2.

**Extended Data Fig. 9:**
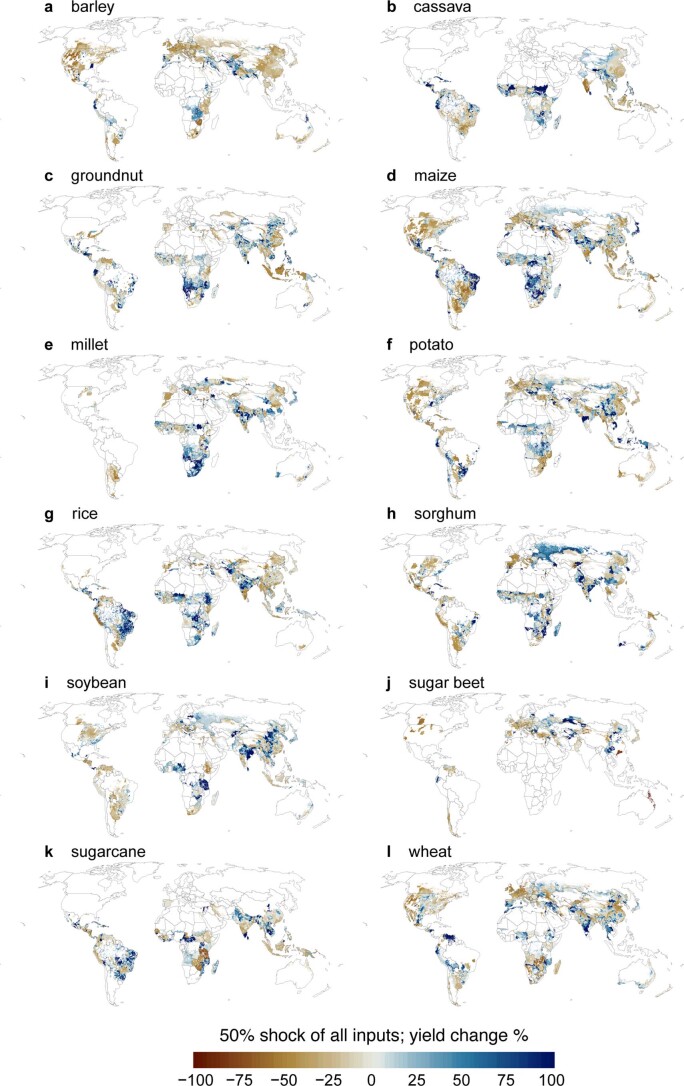
Crop yield changes across all crops after a 50% shock in all inputs. Results are shown for each crop separately: a. barley, b. cassava, c. groundnut, d. maize, e. millet, f. potato, g. rice, h. sorghum, i. soybean, j. sugar beet, k. sugarcane, and l. wheat. Here both decreased and increased yields are shown, otherwise the figure is similar to Fig. 3.

Interestingly, we found a large variation in yield response to shock between the areas of low agricultural input rates (see Agri.Input.Shock -explorer), and in some agricultural areas the shocks increased the yields (Extended Data Figs. 8 and 9). These areas—located mostly in sub-Saharan Africa and South Asia—might indicate yield gap areas; that is, in other parts of the climate bin in question better yields are achieved with similar or smaller input (Discussion). The areas also match relatively well with yield gap studies (for example, Mueller et al.^12^). For example, in sub-Saharan Africa synthetic fertilizers are not used in large quantities due to high prices. In addition, acidic soil conditions and imbalances in N and P fertilizer ratios prevent them from reaching their full potential^13,14^. Best yield results require stoichiometrically balanced fertilizers coupled with adequate water supply and soil modification using machinery. When an agricultural input shock is modelled in an imbalanced system, the results can reflect more balanced conditions and thus increased yields. However, this work has not specifically examined any additional factors or causes contributing to increased yield.

### Shock effects by climate bins

**Fig. 4:**
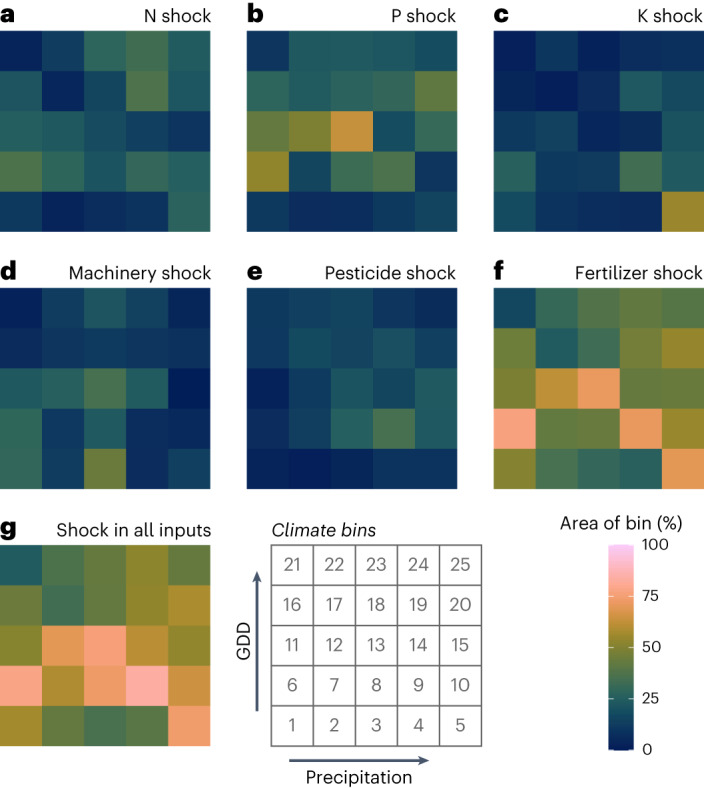
Extent of maize yield reductions due to a 50% shock across inputs. **a**–**g**, Results for each shock: N (nitrogen) shock (**a**); P (phosphorous) shock (**b**); K (potassium) shock (**c**); machinery shock (**d**); pesticide shock (**e**); fertilizer shock (**f**); shock in all inputs (**g**). Impact is shown as the percentage of a climate bin area where the maize yield, after a 50% shock, was at least 10% lower than the original yield. Results for all crops are shown in Agri.Input.Shock -explorer: http://193.166.24.46:3838/shock_shiny/.

To examine the impacts of input shocks in relation to each climate bin, scenario results of all grid cells within each climate bin of a crop were aggregated, focusing on yield decreases. We found clear differences between the climate bins on how the shocks impact on yields: in case of maize, for example, climate bins 6, 9, 11, 12 and 13 (corresponding to temperate climate) seem to respond heavily to P shock, with bin 5 to K shock and bin 3 to machinery shock (Fig. 4). Most climate bins experience yield decrease in a larger area when all inputs have a shock, and in many climate bins the fertilizer shock effect is somewhat similar to the shock in all inputs. At the same time, there are some climate bins, such as 21, that do not see a large yield decrease with any shock type. For many crops, the shock in pesticide inputs has little effect on yield decrease (Fig. 4).

There are similarities and differences between the crops in their yield decrease responses to the different shock scenarios when examined over climate bins (see Agri.Input.Shock -explorer). For most crops, the shock in all fertilizers results in a decrease in yields rather similar to that caused by the shock in all inputs, emphasizing the importance of fertilizers. These findings are in line with an existing study by Pradhan et al.^15^ who studied different agricultural management interventions needed to close yield gaps and determined that fertilizer application and soil quality management were the most important interventions, and pesticides application, along with other strategies were less important on a global level.

Over all the 12 crops, there is no clear pattern detected in the shock-induced yield decline related to the climate bin variables (Agri.Input.Shock -explorer). In individual crops, however, some clustering of heavily affected bins can be seen: in the case of maize, climate bins 6, 8, 9, 12, 13 and 14 share relatively close temperature and precipitation conditions and are also most heavily affected by the shock in all inputs (Fig. 4).

### Changes in production

**Fig. 5:**
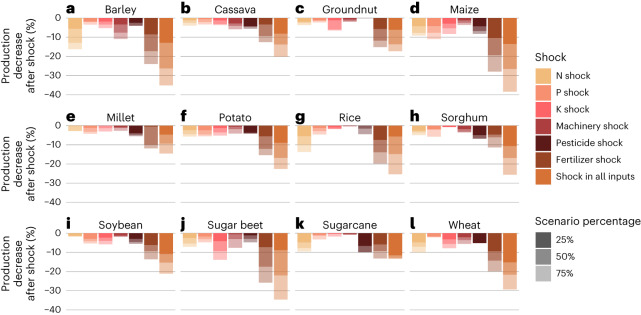
The effects of the different shock scenarios on global production. **a**–**l**, Results for each crop: barley (**a**); cassava (**b**); groundnut (**c**); maize (**d**); millet (**e**); potato (**f**); rice (**g**); sorghum (**h**); soybean (**i**); sugar beet (**j**); sugarcane (**k**); wheat (**l**). Here the production effect is calculated as the product of yield effect and harvested area. The shades indicate the effect of different shock scenario percentages (25%, 50%, 75%).

To account for varying harvested areas across global croplands, we converted the shock scenario results from yields to production volumes by multiplying crop-specific yields (tonnes per hectare (t ha^−1^)) with their harvested area (ha) for each shock scenario. Then we compared these with the original production (t), including both yield decreases and increases. The shock increase did not have a linear effect on production change: for example, in the case of wheat, a 25% shock in all inputs decreased global production by 15%, while a 50% shock decreased the production by 20% (Fig. 5l).

The crop most affected in global production by a shock in all agricultural inputs is maize, where production declines over 25% with 50% shock and nearly 40% with a 75% shock (Fig. 5d). This might be because global maize production is highly optimized with the highest use rate of synthetic fertilizers of all cereals^16^ and therefore more susceptible to decreases in fertilizer inputs. The largest production decreases by shocks in individual agricultural inputs occur in barley, rice and wheat by N rate, and in sugar beet by K rate. Despite not showing considerable effects on overall yields (Fig. 4 and Agri.Input.Shock -explorer), pesticides seem to have a substantial effect on, for example, sugarcane production on the global scale.

**Fig. 6:**
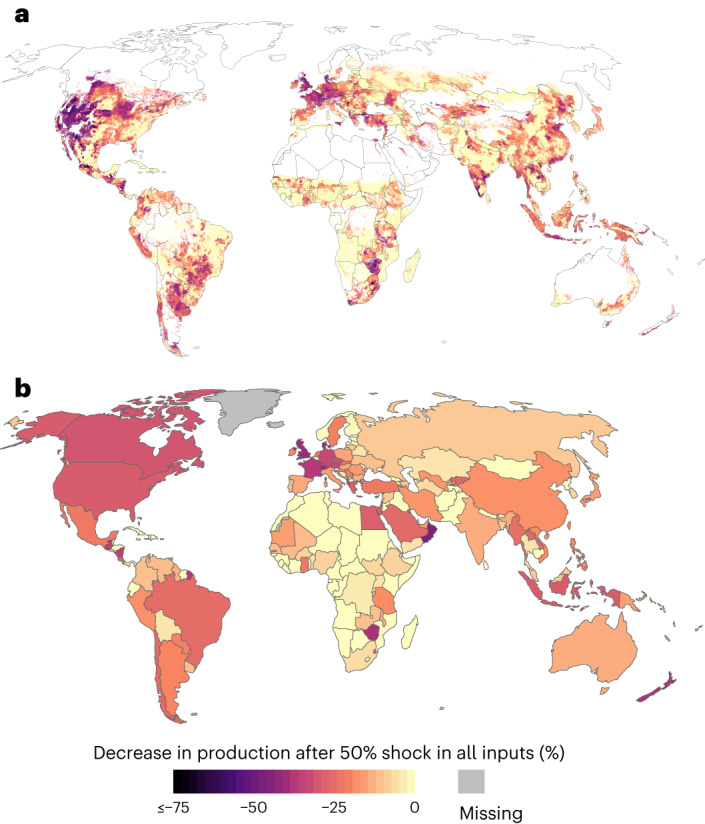
Change in total production of the 12 analysed crops after a 50% shock in all inputs. **a**,**b**, Results on grid cell level (**a**) and by country (**b**). Same colour scale for both maps. Results for all scenarios are shown in Agri.Input.Shock -explorer: http://193.166.24.46:3838/shock_shiny/.

To assess which food production areas and countries would be most impacted by the input shocks, we mapped the cell-wise impacts of shock scenarios (Fig. 6 and Agri.Input.Shock -explorer). Notable decreases in crop production can be seen in many important agricultural regions, such as the United States, Argentina, Western Europe and Southern Africa as well as parts of China and Thailand (Fig. 6a). Country-wise, the largest relative decreases in production (>50% reductions) are experienced in Denmark, Oman, the UK, New Zealand and Saudi Arabia (Fig. 6b). Of the five current top producers of all the studied 12 crops (Brazil, China, India, Thailand and the United States), the United States experienced the largest decline in production, −28%. The largest absolute production decline occurs as well in the United States, where production would fall as much as 140 million tonnes, followed by Brazil with a 114 million-tonne decline. Many countries in Africa and, for example, Finland and the Baltics would suffer relatively little of this kind of input shock (Fig. 6b).

Finally, we calculated the change in production for the most important export countries of each major crop^17–19^ after a 50% shock in all agricultural inputs (Table 1). The production decreases for the major exporting countries are substantial and would likely result in food security issues in the countries heavily dependent on imports; the Middle East and North African region are dependent on Western Europe for wheat, Central America depends on the United States for maize and West Africa depends on rice from Thailand^17,18^. Global food security would be further threatened by ensuing food price increases and possible export restrictions. More research is needed to fully understand how the input shocks would propagate in the interconnected food trade network.

**Table 1:** Impact of a 50% shock in all agricultural inputs on maize, rice, soybean and wheat production in the most important exporting countries^17–19^

Maize (production change, %)		Rice (production change, %)		Soybean (production change, %)		Wheat (production change, %)	
Argentina	−47	India	−7	Argentina	−22	Australia	−1
Brazil	−36	Thailand	−8	Brazil	−13	Canada	−29
China	−16	USA	−27	USA	−22	Germany	−48
France	−32	Vietnam	−29			France	−39
USA	−34					Russia	−19
						USA	−11

## Discussion

The relationships between the use of agricultural inputs and crop yields are complex and difficult to assess with a single process-based model. To overcome this bottleneck, we developed a machine learning model using random forest algorithms that allowed us to predict the changes in crop yield in the face of single and combined agricultural input shocks. Although some existing studies have also examined the effects of agricultural input shocks or reductions, those have been much more limited in terms of geographical extent and/or inputs considered than our study. Our shock scenario of a 25% shock in all inputs resulted in decreases of −22%, −3% and −20% in wheat production in China, the United States and the EU, respectively, which are rather well in line with the findings of Beckman et al.^5^ who modelled the reduction of pesticides by 50% and fertilizers by 20% and found changes of −33%, +3% and −33%, respectively.

In a more local study, Jansik et al.^9^ found that a total shock in the input of farm chemicals, fertilizers and pesticides could reduce yields by 10–40% (crops not specified). In our scenarios, a 75% shock in all inputs reduced the yields of Finnish wheat, barley and potato by a maximum of 45%. Jansik et al.^9^ also conclude that the effects of pesticide shocks would be severe: grain yields could decrease by 30% and potato yields by 50% or more. In our study, pesticide shocks had a low effect globally, but in our 75% pesticide shock scenarios for Finland, barley, potato and wheat yields decreased moderately: 12%, 14% and 25%, respectively. Due to the finite resources of P, few other national or larger-scale studies have estimated the impacts of shock on its availability or price increases. For example, O’Hara et al.^20^ simulated using an economic model that −11% and −26% change in application rates of phosphate and potash resulted in a 14% reduction in maize production. In our somewhat comparable shock scenarios, a 25% P shock and a 25% fertilizer shock, maize production in India decreased by 4% and 8%, respectively.

### Model performance

**Extended Data Fig. 10:**
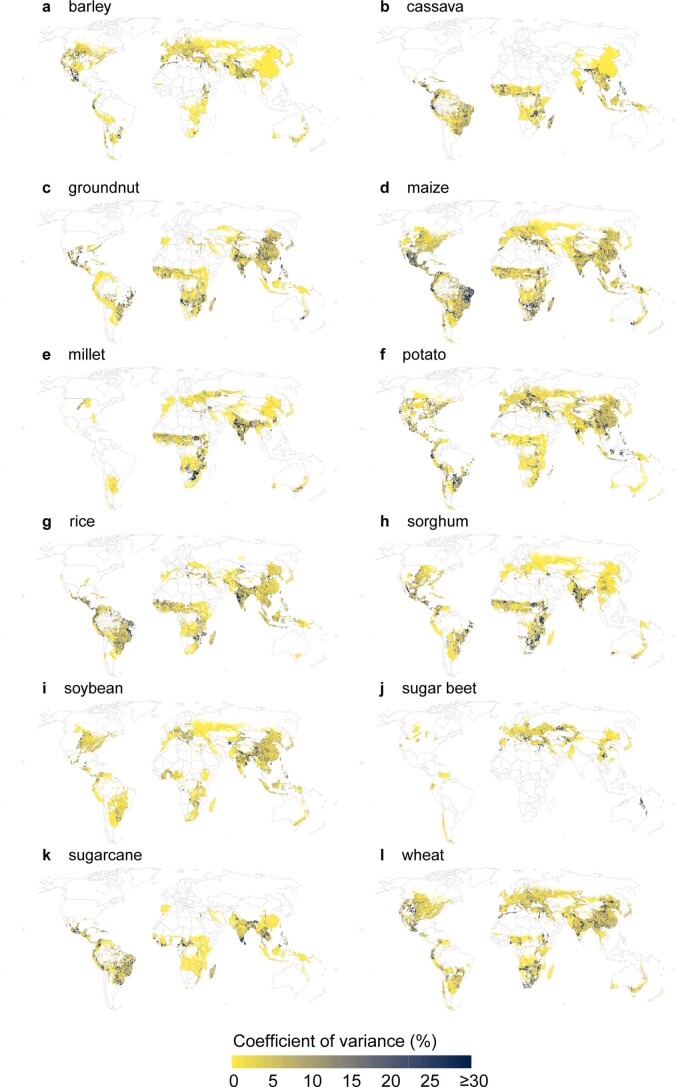
Coefficient of variance (c.v.) of the crop yield predictions for the baseline model runs. Results are shown for each crop separately: a. barley, b. cassava, c. groundnut, d. maize, e. millet, f. potato, g. rice, h. sorghum, i. soybean, j. sugar beet, k. sugarcane, and l. wheat. The c.v.s are calculated across the 25 baseline model runs (see Methods) for each location (grid cell) where the crop in question is grown.

Most (95%) of the random forest models for crops and climate bins had NSE scores above 0.65, and for 79% of the models the NSE scores were even above 0.75 (Extended Data Fig. 3), indicating good or very good model performance^21^, respectively. There was, however, considerable variation between crop–climate bin combinations, with some performing better than others (Extended Data Fig. 3; see also uncertainty results in Methods and Extended Data Fig. 10). For lower-performing climate bins, it is possible that the input data are not diverse enough to produce good models or that climate or other factors play a more important role than the agricultural inputs we studied. Our NSE values align with the results of Jeong et al.^22^, who apply random forest models to predict global wheat yields. They note, however, that their good results may be a product of spatial autocorrelation between data points in similar political units. Ferraciolli et al.^23^ show that spatial autocorrelation indeed increases overfitting in their yield model—that they applied for sugarcane—and that it underestimates the error of the model. Similarly to Ferraciolli et al.^23^, we aimed to minimize the effects of overfitting and spatial autocorrelation on model evaluation by dividing the training and testing data into grids to keep adjacent cells in different groups to make sure that the model predictions were truly based on relationships in the data (Methods).

### Study limitations and way forward

Most of the input and yield data are from the same period, with averages around the year 2000 (Supplementary Table 2). We assumed that the relationships between the inputs and yield have remained quite similar and changes are slow and that the results also apply to the current-day situation. Yet another aspect that could have affected the model performances is some details of the model input data: for a wheat climate bin with 28,000 observations, there are only 30 unique values for N rate or P rate, and furthermore, many of the fertilizer rates are known only at subnational or county level^12^. The low level of details in the data could increase spatial autocorrelation, and more detailed data would improve our model’s accuracy and prediction power (Methods).

We found that the largest yield decreases were observed for high yields, while lower yields tended not to be affected negatively by the shocks (Fig. 1). Higher yields likely have a higher dependency on agricultural inputs than lower yields, and the variation of high yields can be more readily explained by variation in agricultural input use. This result confirms the findings from an earlier study projecting a conversion to organic agriculture with no synthetic fertilizer use and where the currently high-yielding areas were most affected as well^24^.

We also found that some yields increased after input shock (Fig. 1 and Extended Data Figs. 5 and 6). Methodologically, it is important to note that instead of indicating an actual yield increase in the specific area due to decreasing inputs, this means there is another similar area with smaller inputs but a higher yield. In the Results section, we discuss whether these indicate yield gap areas. Furthermore, yet another possible or partial explanation can be found inherently in the random forest algorithm; it does not perform as well in the extreme ends or beyond the dataset where extrapolation is needed^10,25^. The model cannot, for example, accurately predict smaller yield values than what it was trained on. The crop yield and agricultural input data are less detailed in the low-yielding areas of the dataset and thus do not allow the model to perform so well in these areas. In addition, the long-term risks to yields are not captured by the model. For example, a short-term shock in synthetic P could be buffered by soil P stocks, thus showing a smaller impact in the short term. However, the model does not capture possible long-term impacts due to changes in soil P availability. These are, however, out of the scope of this study as our main aim was to assess the short-term threats of agricultural input shocks and yield decreases, and the inclusion of these features was left for future studies.

Finally, including additional agricultural inputs not studied here, for example, seeds, could provide further understanding of the impacts of potential shocks and trade disturbances. We also acknowledge that the flow of agricultural inputs is intimately related to economics and trade. From the total production perspective, general equilibrium models can efficiently allocate resources and direct investments in resource shock mitigation. Yet, the economic analysis was outside our focus and thus not included in this study, which concentrated on the magnitude of the impacts of shocks, regardless of their cause. One idea is to use gravity models of trade to estimate the volume of flows of agricultural inputs between places by considering productivity and various costs^26^. Furthermore, the reserve stocks of the agricultural inputs that countries keep as a buffer against disturbances in import flows can impact the way countries are affected. However, to our knowledge, no such data are available globally, and therefore we could not include those in our analysis.

## Concluding remarks

We found that the input shocks would hit the high-yielding ‘breadbaskets’ particularly hard and thus have a considerable impact on global food security. Although our results do not reveal any single agricultural input as being the most influential in shock yield decreases across all crop–climate bin combinations, most of the decrease was attributed to shocks in synthetic fertilizers. To increase food security and resilience, areas with a high degree of synthetic fertilizer use—and particularly those depending on imported fertilizers—combined with high potential yield losses due to shocks should seek to replace them with more sustainable and local organic fertilizers. Our results can be used to evaluate regional food security more comprehensively and, together with other information, help to identify areas under risk. All in all, agricultural inputs and their availability should be considered with more emphasis when constructing a more resilient and sustainable global food system.

## Methods

### Crop yield data

The availability of global gridded crop yield and agricultural input data limited the selection of food crops for the analysis. Twelve globally important food crops were selected for analysis for which all necessary data were found: barley, cassava, groundnut, maize, millet, potato, rice, sorghum, soybean, sugar beet, sugarcane and wheat.

Yield data (t ha^−1^) were sourced from Monfreda et al.^27^. They represent the average yields between 1997 and 2003, thus minimizing the effect of interannual yield variability. For production calculations, harvested area (ha) from the same dataset was used (Supplementary Table 2).

### Agricultural input data

For the gridded fertilizer data, we used the synthetic fertilizer application, including N, P and K fertilizers, in Mueller et al.^12^. We chose to concentrate only on synthetic fertilizers as they are often imported from elsewhere and thus more prone to, for example, shocks in trade networks. We also included the non-mineral fertilizer layer from the EarthStat database (West et al.^28^) to represent the locally available fertilizers, but this was kept constant in all scenarios. For machinery use, we used US Department of Agriculture data of International Agricultural Productivity^29^ per 1,000 ha of cropland having data of agricultural machinery that were aggregated across multiple years to an average for each country and transformed into a raster. These data include the country-level use of major farm equipment (four-wheel riding tractors, two-wheel pedestrian tractors, power harvester–threshers and milking machines) presented in total metric horse-power. If the data source did not have machinery data for a particular country, the average of the continent was used. The machinery data are not crop specific but rather proxy measures of the degree of agricultural mechanization used in the area. We also included a proxy for how intensively the crop production is based on human labour by including national-level data on people working in the agriculture sector (FAOSTAT^11^) per 1,000 ha of cropland. This was kept constant in all scenarios.

Pesticide data from Maggi et al.^30^ consist of application rate data of the 20 most used pesticides for selected crops (wheat, maize, rice and soybean) as well as different crop classes. For the eight other crops, the aggregate class Other was used, as classified by Maggi et al.^30^. The pesticide data were given as high and low estimates, but for our analysis, a mean estimate was calculated from these. As it was not feasible to add all the 20 pesticides (per crop) individually to the model, we divided the pesticides into four groups: Herbicides, Insecticides, Fungicides and Others. Furthermore, due to the varying application rates of different pesticides within each group, in terms of weight per hectare, we needed to rescale the application rates. We therefore rescaled each pesticide application rate so that the application rate of each grid cell was divided with the 97.5 percentile application rate (of a given pesticide) found globally. This resulted in a new gridded application rate dataset for each pesticide, the rate varying from 0 to 1 (values above 97.5 percentile were given value 1). These rescaled pesticide grids within each group were then summed together. This resulted in four ‘pesticide’ inputs. This way we were able to decrease the amount of pesticide variables to just four, while retaining the weight of each individual pesticide and also separating the key pesticide groups. Straightforward summing of individual pesticide applications rates within each group would have masked the active ingredients that are only needed in smaller quantities. For future development of the model, a more sophisticated way of dealing with the pesticides could be explored.

We included a statistic for irrigation in our model, as irrigated crops have higher yields^31^. Irrigation also enhances the effect of fertilization: the same fertilizer input produces higher yields on irrigated crops than on non-irrigated, based on empirical studies by, for example, Di Paolo and Rinaldi^32^ as well as yield modelling results by Mueller et al.^12^. Even in a situation of agricultural input shocks, the infrastructure for irrigation would remain unchanged and could potentially alleviate the effects of decreased inputs. Crop-specific irrigated and rainfed harvested area was sourced from Portmann et al.^33^, and for the analysis we transformed it to the share of harvested area under irrigation (%).

### Soil data

We added altogether three soil parameters to the machine learning model to represent soil quality. We included gridded soil P from McDowell et al.^34^ and soil N from SoilGrid v2^35^ for the topsoil (0–30 cm) to represent the natural availability of P and N, respectively, to the crops. Furthermore, we used soil organic carbon as it is shown to impact yields^36^ and to be a good indicator for soil degradation^37^. For that we used organic carbon density (hg m^−3^) from SoilGrid v2^35^ for the topsoil (0–30 cm). We aggregated this and soil N from 1 km to 5 arcmin resolution (using mean value over each aggregated grid cell) and converted the unit to t ha^−1^.

### Climate bins

To control for the yield variation caused by climate, we divided each crop into climate bins to study the variation in yield caused only by the agricultural inputs. For example, crops in Finland are compared to crops in similar climate bins in Canada, Russia and China to capture the relationships between inputs and yield in their respective climates. The climate bin method has been used successfully before in global agricultural production analyses^12,38,39^. Johnston et al.^38^ and Licker et al.^39^ use GDD and the soil moisture index to construct the climate bins, while Mueller et al.^12^ use GDD and precipitation and divide the bins by equal harvested area. We followed the approach by Mueller et al.^12^ and used GDD and precipitation. We created crop-specific climate bins and wanted to ensure that each climate bin would have an equal amount of data points (that is, grid cells with cropland of a crop in question), and therefore could not use any readily available dataset.

To create crop-specific climate bins with equal amounts of data points in each bin, we used daily temperature and precipitation over 21 years (1990–2010) from AgMERRA^40^. AgMERRA is a climate forcing dataset based on the US National Aeronautics and Space Administration (NASA) Modern-Era Restrospective Analysis for Research and Applications (MERRA), but is corrected especially for agricultural applications. To estimate the GDD, we used the approach from existing studies^12,39,41^ and summed the temperature over the days with higher temperatures than baseline temperature (Supplementary Table 3). Furthermore, we used the cut-off temperature (as in Grigorieva et al.^42^) to represent the temperature ceiling that a crop can benefit from; that is, if a temperature of a day was over the cut-off temperature, the cut-off temperature was used instead. We estimated the GDD for each year over the 21 years and used an average GDD over these years for the climate bins. For precipitation, we used an average annual cumulative precipitation. For each crop area (that is, the area on which a crop is grown globally), GDD and precipitation were divided into five quantiles to group the crop area into 25 different climate bins (see example for wheat in Extended Data Fig. 1). While the existing studies have divided the world into 100 climate bins^12,38,39^, we used only 25 bins to ensure enough data points in each climate bin.

### Random forest model

Preliminary examination of the agricultural input data revealed that within a climate bin, the relationship between the agricultural input and crop yields was not linear. Furthermore, some of the agricultural inputs correlated with each other (for example, areas with high fertilizer input often also have high pesticide input). A machine learning approach was thus selected, as it can handle nonlinear relationships as well as predictor interactions and, furthermore, it is able to process many different types of dataset with minimal intervention^43^.

Random forest^10^ is a machine learning algorithm based on classification and regression trees^44^. Here the trees are constructed with bootstrapped data, that is, a random subset of the data with resampling. Within each node of the tree, a defined number of randomly selected parameters is used to split the node so that the weighted variance is minimized. Hundreds or thousands of trees are constructed like this, making them uncorrelated and preventing overfitting. The final output of the random forest regression is the average of all the output values of the individual uncorrelated trees in the forest. Random forest can be adjusted with different hyperparameter values^45^. A larger number of trees grown (*ntree*) improves the accuracy of the random forest but increases the required computing power. The number of parameters used to split the node (*mtry*) is usually set to *p*/3 in case of regression, where *p* is the number of variables in the model. The third hyperparameter value is *nodesize* which is the minimum number of observations remaining in the tree’s terminal (leaf) node. The default value for regression trees is 5. Smaller *nodesize* values lead to deeper trees, because more nodes are needed to reach the small terminal nodes. The random forest default values have been shown to produce good results^46^.

Random forest has been successfully used for yield predictions with smaller-scale climate, irrigation and satellite data^47–51^. It has also been used in agricultural modelling to help select the important variables for constructing another type of model^52^.

Jeong et al.^22^ compare the random forest algorithm in global yield predictions with multiple linear regression. They find that in global wheat yield prediction as well as smaller-scale maize and potato yield prediction, random forests outperform multiple linear regression in prediction accuracy. However, Jeong et al.^22^ use mostly meteorological and geophysical variables and only one agricultural input (N). In predicting yield variability in time and space, Feng et al.^53^ and Leng and Hall^43^ also find that random forest performs better than regression or process-based models, but they only use climate data. To our knowledge, no random forest analyses have been done with global, trade-dependent agricultural input data.

We constructed the random forest models for each of the 12 crops and their 25 climate bins. The agricultural input and yield data were assigned into their respective climate bins and transformed into data frames, where each row consisted of a 5 arcmin grid cell and all its agricultural input and yield data.

Random forest regression was then performed with R package randomForest (4.7-1.1) (ref^. 54^) on each climate bin and crop individually, using crop yield as a dependent variable and agricultural inputs with soil organic carbon as independent variables. To minimize the effects of overfitting and spatial autocorrelation in the evaluation of the models, the data in each climate bin were divided randomly between training and testing data. One degree (60 arcmin) grid was imposed over the 5 arcmin resolution data to divide it into square sets of 12 × 12 grid cells. Of these sets 75% were used for training and 25% for testing, thus limiting the number of consecutive 5 arcmin cells in each training–testing iteration.

The training data were used to construct the forest with default hyperparameter values (1,000 trees, a minimum of 5 terminal nodes and using 2 parameters to split the nodes). Default values were used for all forests for all crops, as preliminary testing with hyperparameter tuning showed little or no improvement to model performances, and the default values have been shown to produce good results^46^.

### Agricultural input shock scenario setting

The constructed models allowed us to predict the effect of different agricultural input shock scenarios on crop yields. We developed the following scenarios: individual input shocks (N shock, P shock, K shock, machinery shock, pesticide shock), shock in all fertilizers and shock in all inputs. Three degrees of shock severity were used for each scenario: 25%, 50% and 75% decreases in inputs including in a scenario.

For each climate bin and crop combination, forest construction and scenario prediction were iterated 25 times with results saved for each iteration. We were thus able to calculate prediction variances between iterations, improving the estimation of model stability^43^. The scenario results were compared against the baseline model run.

In our study, random forest regression predicts the shock scenario yields by ‘selecting’ observations in the same climate bin where input use is in the baseline similar to scenario use. Decreased scenario shock yields indicate that within the climate bin in question, the baseline yields were only attainable with original input values. Increased yields after scenario shocks mean that in the same climate bin, similar or better yields are possible with less commercial agricultural inputs. It should also be noted that the model cannot take into account potential other effects of or adaptations on the shocks, such as reallocation of crops or labour.

All analyses and calculations were performed using R software version 4.0.4^55^ using R Studio. The code is available in GitHub: https://github.com/ahvoa/shock_pub. All the raster files from the scenario results can be accessed in the Zenodo data repository (10.5281/zenodo.8381197). Due to the large amount of data generated in the analyses, Agri.Input.Shock -explorer using R Shiny app was created to view and discuss the results. The Agri.Input.Shock -explorer is available at http://193.166.24.46:3838/shock_shiny/. The code for the Agri.Input.Shock -explorer is available for local installation at the GitHub repository mentioned above, in case the above link stops working at some stage.

### Model performance and validation

Model performance was estimated by comparing the model predictions of the testing data to the known original yields using root mean square error (r.m.s.e., see Agri.Input.Shock -explorer; and NSE, Extended Data Fig. 3). For most of the crops and climate bins, the NSE scores are above 0.65 (Extended Data Fig. 3), indicating that the models have good (NSE > 0.65) or very good (NSE > 0.75) simulation results and predicting power^21^. The scores for sugar beet and barley seem to be especially high, while for cassava the climate bins with high precipitation and groundnut climate bins with low GDD have relatively poor scores. Scenario predictions from these poorly scored climate bins should be examined with caution. More visualizations of model performances and behaviours can be viewed in the Agri.Input.Shock -explorer (http://193.166.24.46:3838/shock_shiny/).

Furthermore, we validated the mean country-level yields of the baseline runs against the reported yields in FAOSTAT for the years 1997–2003^11^ weighted with modelled production. The comparison showed that the baseline yields from the model are mostly very well in line with the reported yields (*R*^2^ > 0.8 for all the crops; Supplementary Table 1 and Extended Data Fig. 5). The modelled yields for the baseline are mapped in Extended Data Fig. 4.

In addition, we validated the model performance by setting a control scenario where all the inputs were set to zero. The results of this control scenario did not show zero yields but instead an appreciable yield confirming the robustness of the model (Extended Data Figs. 6 and 7).

### Uncertainty

A full-scale uncertainty analysis could not be conducted for a lack of uncertainty measures in most datasets used in our work and capability of the methods deployed here to account for other sources of uncertainty. However, to assess the uncertainty related to the model inputs, for each crop and climate bin we calculated the model outputs for the baseline model run 25 times, while randomly sampling the data that go into the training and testing sets. This approach allowed us to quantify the effects that the variability in model inputs have on the modelled crop yields. We then calculated the coefficient of variance (c.v.) of the predicted crop yields for each grid cell to understand where their variability, and hence the uncertainty, is the largest.

The analysis shows that for most of the global crop-growing areas, the uncertainty is relatively low, as the c.v. of the crop yield predictions remains below 10% (Extended Data Fig. 10). This means that the standard deviation of the yield predictions is less than 10% of the average prediction. Relatively large variations are found, for example, in the eastern coast of Africa (sorghum), India (millet) and Southeast Asia (sugarcane), while Europe generally shows relatively low variability (Extended Data Fig. 10).

### Reporting summary

Further information on research design is available in the Nature Portfolio Reporting Summary linked to this article.

### Supplementary information

Supplementary Information Supplementary Tables 1–3.

Reporting Summary

## Data Availability

All the input data are openly available from the sources mentioned in Methods. All the output raster files from the scenario results can be accessed in the data repository: 10.5281/zenodo.8381197.

## Code availability

The analysis codes are available in GitHub: https://github.com/ahvoa/shock_pub.

## References

[1] Yu Q (2020). A cultivated planet in 2010—Part 2: The global gridded agricultural-production maps. Earth Syst. Sci. Data.

[2] Hebebrand, C. & Laborde, D. High fertilizer prices contribute to rising global food security concerns. *IFPRI Blog* https://www.ifpri.org/blog/high-fertilizer-prices-contribute-rising-global-food-security-concerns (2022).

[3] Marchand P (2016). Reserves and trade jointly determine exposure to food supply shocks. Environ. Res. Lett..

[4] Bonilla-Cedrez C, Chamberlin J, Hijmans RJ (2021). Fertilizer and grain prices constrain food production in sub-Saharan Africa. Nat. Food.

[5] Beckman, J., Ivanic, M., Jelliffe, J. L., Baquedano, F. G. & Scott, S. G. *Economic and Food Security Impacts of Agricultural Input Reduction Under the European Union Green Deal’s Farm to Fork and Biodiversity Strategies*. Economic Brief No. 30, (USDA Economic Research Service, 2020).

[6] Jacquet F (2022). Pesticide-free agriculture as a new paradigm for research. Agron. Sustain. Dev..

[7] Dall’Erba S, Chen Z, Nava NJ (2021). U.S. Interstate trade will mitigate the negative impact of climate change on crop profit. Am. J. Agric. Econ..

[8] Ferguson SM, Gars J (2020). Measuring the impact of agricultural production shocks on international trade flows. Eur. Rev. Agric. Econ..

[9] Jansik, C. et al. *Maatalouden tuotantopanosten saatavuuden riskit: Kriiseihin varautuminen ruokahuollon turvaamisessa*. 98 http://urn.fi/URN:ISBN:978-952-380-300-8 (2021).

[10] Breiman L (2001). Random forests. Mach. Learn..

[11] *Food and Agriculture Data (FAOSTAT)* (FAO, 2023); https://www.fao.org/faostat/

[12] Mueller ND (2012). Closing yield gaps through nutrient and water management. Nature.

[13] Burke WJ, Jayne TS, Black JR (2017). Factors explaining the low and variable profitability of fertilizer application to maize in Zambia. Agric. Econ..

[14] van der Velde M (2014). African crop yield reductions due to increasingly unbalanced nitrogen and phosphorus consumption. Glob. Change Biol..

[15] Pradhan P, Fischer G, Velthuizen H, Reusser DE, Kropp JP (2015). Closing yield gaps: how sustainable can we be?. PLoS ONE.

[16] *IFASTAT* (International Fertilizer Association, 2023); https://www.ifastat.org/

[17] d’Amour CB, Wenz L, Kalkuhl M, Steckel JC, Creutzig F (2016). Teleconnected food supply shocks. Environ. Res. Lett..

[18] Headey D (2011). Rethinking the global food crisis: the role of trade shocks. Food Policy.

[19] Puma MJ, Bose S, Chon SY, Cook BI (2015). Assessing the evolving fragility of the global food system. Environ. Res. Lett..

[20] O’Hara JK, Mulik K, Gurian-Sherman D (2015). Agricultural production impacts of higher phosphate fertilizer prices. J Int. Agric. Trade Dev..

[21] Moriasi DN (2007). Model evaluation guidelines for systematic quantification of accuracy in watershed simulations. Trans. ASABE.

[22] Jeong JH (2016). Random forests for global and regional crop yield predictions. PLoS ONE.

[23] Ferraciolli MA, Bocca FF, Rodrigues LHA (2019). Neglecting spatial autocorrelation causes underestimation of the error of sugarcane yield models. Comput. Electron. Agric..

[24] Barbieri P (2021). Global option space for organic agriculture is delimited by nitrogen availability. Nat. Food..

[25] Segal, M. R. *Machine Learning Benchmarks and Random Forest Regression* (2004); https://escholarship.org/uc/item/35x3v9t4

[26] Nava NJ, Ridley W, Dall’erba S (2023). A model of the U.S. food system: what are the determinants of the state vulnerabilities to production shocks and supply chain disruptions?. Agric. Econ..

[27] Monfreda C, Ramankutty N, Foley JA (2008). Farming the planet: 2. Geographic distribution of crop areas, yields, physiological types, and net primary production in the year 2000. Glob. Biogeochem. Cycles.

[28] West PC (2014). Leverage points for improving global food security and the environment. Science.

[29] U.S. Department of Agriculture Economic Research Service. *International Agricultural Productivity* (USDA, 2023); https://www.ers.usda.gov/data-products/international-agricultural-productivity/

[30] Maggi, F., Tang, F. H. M., Cecilia, D. & McBratney, A. PEST-CHEMGRIDS, global gridded maps of the top 20 crop-specific pesticide application rates from 2015 to 2025. *Sci. Data* **6**, 170 (2019). 10.1038/s41597-019-0169-4 PMC6761121 31515508

[31] Lobell DB, Cassman KG, Field CB (2009). Crop yield gaps: their importance, magnitudes, and causes. Annu. Rev. Environ. Resour..

[32] Di Paolo E, Rinaldi M (2008). Yield response of corn to irrigation and nitrogen fertilization in a Mediterranean environment. Field Crops Res..

[33] Portmann FT, Siebert S, Döll P (2010). MIRCA2000—global monthly irrigated and rainfed crop areas around the year 2000: a new high-resolution data set for agricultural and hydrological modeling. Glob. Biogeochem. Cycles.

[34] McDowell RW, Noble A, Pletnyakov P, Haygarth PM (2023). A global database of soil plant available phosphorus. Sci. Data.

[35] Poggio L (2021). SoilGrids 2.0: producing soil information for the globe with quantified spatial uncertainty. SOIL.

[36] Oldfield EE, Bradford MA, Wood SA (2019). Global meta-analysis of the relationship between soil organic matter and crop yields. SOIL.

[37] Obalum SE, Chibuike GU, Peth S, Ouyang Y (2017). Soil organic matter as sole indicator of soil degradation. Environ. Monit. Assess..

[38] Johnston M (2011). Closing the gap: global potential for increasing biofuel production through agricultural intensification. Environ. Res. Lett..

[39] Licker R (2010). Mind the gap: how do climate and agricultural management explain the ‘yield gap’ of croplands around the world?. Glob. Ecol. Biogeogr..

[40] Ruane AC, Goldberg R, Chryssanthacopoulos J (2015). Climate forcing datasets for agricultural modeling: merged products for gap-filling and historical climate series estimation. Agric. For. Meteorol..

[41] Hodges, T. *Predicting Crop Phenology* (CRC, 1990).

[42] Grigorieva EA, Matzarakis A, Freitas CR (2010). Analysis of growing degree-days as a climate impact indicator in a region with extreme annual air temperature amplitude. Clim. Res..

[43] Leng G, Hall JW (2020). Predicting spatial and temporal variability in crop yields: an inter-comparison of machine learning, regression and process-based models. Environ. Res. Lett..

[44] Breiman, L. *Classification and Regression Trees* (Routledge, 2017); 10.1201/9781315139470

[45] Probst P, Wright MN, Boulesteix A-L (2019). Hyperparameters and tuning strategies for random forest. WIREs Data Min. Knowl. Discov..

[46] Fernández-Delgado M, Cernadas E, Barro S, Amorim D (2014). Do we need hundreds of classifiers to solve real world classification problems?. J. Mach. Learn. Res..

[47] Chlingaryan A, Sukkarieh S, Whelan B (2018). Machine learning approaches for crop yield prediction and nitrogen status estimation in precision agriculture: a review. Comput. Electron. Agric..

[48] Everingham Y, Sexton J, Skocaj D, Inman-Bamber G (2016). Accurate prediction of sugarcane yield using a random forest algorithm. Agron. Sustain. Dev..

[49] Fukuda S (2013). Random forests modelling for the estimation of mango (*Mangifera indica* L. cv. Chok Anan) fruit yields under different irrigation regimes. Agric. Water Manage..

[50] Johnson MD, Hsieh WW, Cannon AJ, Davidson A, Bédard F (2016). Crop yield forecasting on the Canadian prairies by remotely sensed vegetation indices and machine learning methods. Agric. For. Meteorol..

[51] Newlands NK (2014). An integrated, probabilistic model for improved seasonal forecasting of agricultural crop yield under environmental uncertainty. Front. Environ. Sci..

[52] Tulbure MG, Wimberly MC, Boe A, Owens VN (2012). Climatic and genetic controls of yields of switchgrass, a model bioenergy species. Agric. Ecosyst. Environ..

[53] Feng P (2018). Impacts of rainfall extremes on wheat yield in semi-arid cropping systems in eastern Australia. Clim. Change.

[54] Liaw, A. & Wiener, M. Classification and regression by randomForest. *R News* **2**, 18–22 (2002).

[55] R Core Team. *R: A Language and Environment for Statistical Computing* (R Foundation for Statistical Computing, 2021).

