# Supplementary material for: Agricultural input shocks affect crop yields more in the high-yielding areas of the world
Source: Nat Food. 2023 Nov 9;4(12):1037–46. doi: 10.1038/s43016-023-00873-z (PMC10727984; doi:10.1038/s43016-023-00873-z)
Supplement: Supplementary file 1 — Supplementary Tables 1–3. [file 43016_2023_873_MOESM1_ESM.pdf]

nature food

Article https://doi.org/10.1038/s43016-023-00873-z

Agricultural input shocks affect crop yields more in the high-yielding areas of the world

In the format provided by the authors and unedited

This supplementary file includes:

Supplementary Tables 1-3

Supplementary Table 1: Model performance at country level. Coefficients of determination (R^2) of a weighted linear regression model (weighted with modelled production of each country) and non-weighted linear regression model for the baseline yields and the reported yields in FAOSTAT (1997-2003) [1]. See scatter plot in Extended Data Fig. 5. The modelled yields for the baseline are mapped in Extended Data Fig. 4.

**Item**	**R^2 (weighted)**	**R^2 (non-weighted)**
Barley	0.994	0.908
Cassava	0.973	0.520
Groundnut	0.960	0.355
Maize	0.998	0.964
Millet	0.968	0.716
Potato	0.953	0.905
Rice	0.997	0.936
Sorghum	0.998	0.853
Soybean	0.992	0.811
Sugarbeet	0.968	0.831
Sugarcane	0.875	0.675
Wheat	0.994	0.940

Supplementary Table 2: Spatial datasets used as input for the model.

Name	Unit	Time period	Spatial resolution	Notes	Reference
Yield	t/ha	2000 (average of census data between 1997–2003)	5 arc-min	Crop-specific	Monfreda et al. [2]
Precipitation and Growing Degree Days (GDD)	mm, °C	Daily data over 1990-2010. GDD: annual average; Precipitation: annual average	15 arc-min, resampled to 5 arc-min	Crop-specific; method adapted from Mueller et al (2012)	AgMERRA data from Ruane et al [3]
Machinery	1000 metric horsepower (CV)	1990–2010	Country level	Universal (i.e., not crop-specific)	USDA [4]
People working in agriculture	Persons	1990–2010	Country level	Universal (i.e., not crop-specific)	FAOSTAT [1]
Mineral fertiliser application rate	kg/ha	2000*	5 arc-min	Crop-specific, mineral fertilisers	Mueller et al. [5]
Nonmineral fertiliser application rate	kg/ha	2000*	5 arc-min	Crop-specific, manure-based fertilisers	West et al. [6]
Pesticide application rate	Original kg/ha, for analysis rescaled by dividing with 97.5th percentile	Estimation for year 2015	5 arc-min	Crop-specific for maize, rice, soybean and wheat, aggregate classes for others	Maggi et al [7]
Share of harvested area under irrigation	%	Around the year 2000	5 arc-min	Crop-specific	MIRCA2000 data from Portmann et al. [8]
Soil P	mg/kg	Varies across the globe	30 arc-sec (ca 1 km) grid aggregated to 5 arc-min	Universal (i.e., not crop-specific)	McDowell et al [9]
Soil N for 0-30 cm topsoil	hg/m3; converted to t/ha	Varies across the globe	1 km grid aggregated to 5 arc-min	Universal (i.e., not crop-specific)	SoilGrids v2 Poggio et al [10]
Soil organic carbon for 0-30 cm topsoil	hg/m3; converted to t/ha	Varies across the globe	1 km grid aggregated to 5 arc-min	Universal (i.e., not crop-specific)	SoilGrids v2 Poggio et al [10]

* From Mueller et al. [5]: “Data represents the year 2000 largely as a collection of data from 1999, 2000. Data for some countries is as old as 1994 or as recent as 2001.”

Supplementary Table 3: Baseline and cutoff temperatures (in °C) to estimate growing degree days (GDD). Baseline temperature is the lowest temperature that is counted for the GDD while cutoff temperature is the highest.

Crop	Variable	Temperature [°C]	Source
wheat	baseline	0	Hodges, 1990 [11]
barley	baseline	1	Hodges, 1990 [11]
potato	baseline	2	Hodges, 1990 [11]
sugar beet	baseline	2	Hodges, 1990 [11]
rice	baseline	5	Hodges, 1990 [11]
cassava	baseline	8	Hodges, 1990 [11]
groundnut	baseline	8	Hodges, 1990 [11]
millet	baseline	8	Hodges, 1990 [11]
maize	baseline	8	Hodges, 1990 [11]
sorghum	baseline	8	Hodges, 1990 [11]
soybean	baseline	8	Hodges, 1990 [11]
sugarcane	baseline	8	Hodges, 1990 [11]
wheat	cutoff	25	Grigorieva et al., 2010 [12]
barley	cutoff	35	Supit et al., 2010 [13]
potato	cutoff	30	Grigorieva et al., 2010 [12]
sugar beet	cutoff	21	Supit et al., 2010 [13]
rice	cutoff	36	Hatfield et al., 2011 [14]
cassava	cutoff	29	FAO and IIASA, 2023 [15]
groundnut	cutoff	32	FAO and IIASA, 2023 [15]
millet	cutoff	32	FAO and IIASA, 2023 [15]
maize	cutoff	30	Grigorieva et al., 2010 [12]
sorghum	cutoff	34	Hatfield et al., 2011 [14]
soybean	cutoff	30	Grigorieva et al., 2010 [12]
sugarcane	cutoff	37	FAO and IIASA, 2023

References

1. FAO. Food and agriculture data (FAOSTAT). (2023).
2. Monfreda, C., Ramankutty, N. & Foley, J. A. Farming the planet: 2. Geographic distribution of crop areas, yields, physiological types, and net primary production in the year 2000. *Global Biogeochemical Cycles* **22**, (2008).
3. Ruane, A. C., Goldberg, R. & Chryssanthacopoulos, J. Climate forcing datasets for agricultural modeling: Merged products for gap-filling and historical climate series estimation. *Agricultural and Forest Meteorology* **200**, 233–248 (2015).
4. USDA U.S. Department of Agriculture Economic Research Service. International Agricultural Productivity. https://www.ers.usda.gov/data-products/international-agricultural-productivity/ (2023).
5. Mueller, N. D. *et al.* Closing yield gaps through nutrient and water management. *Nature* **490**, 254–257 (2012).
6. West, P. C. *et al.* Leverage points for improving global food security and the environment. *Science* **345**, 325–328 (2014).
7. Maggi, F., Tang, F. H. M., Cecilia, D. & McBratney, A. PEST-CHEMGRIDS, global gridded maps of the top 20 crop-specific pesticide application rates from 2015 to 2025. *Scientific Data* **6**, 1–20 (2019).
8. Portmann, F. T., Siebert, S. & Döll, P. MIRCA2000—Global monthly irrigated and rainfed crop areas around the year 2000: A new high-resolution data set for agricultural and hydrological modeling. *Global Biogeochemical Cycles* **24**, (2010).
9. McDowell, R. W., Noble, A., Pletnyakov, P. & Haygarth, P. M. A Global Database of Soil Plant Available Phosphorus. *Sci. Data* **10**, (2023).

10. Poggio, L. *et al.* SoilGrids 2.0: producing soil information for the globe with quantified spatial uncertainty. *SOIL* 7, 217–240 (2021).

11. Hodges, T. *Predicting crop phenology*. (Crc Press, 1990).

12. Grigorieva, E. A., Matzarakis, A. & De Freitas, C. R. Analysis of growing degree-days as a climate impact indicator in a region with extreme annual air temperature amplitude. *Clim. Res.* **42**, 143–154 (2010).

13. Supit, I. *et al.* Recent changes in the climatic yield potential of various crops in Europe. *Agric. Syst.* **103**, 683–694 (2010).

14. Hatfield, J. L. *et al.* Climate impacts on agriculture: Implications for crop production. *Agron. J.* **103**, 351–370 (2011).

15. FAO and IIASA. Global Agro Ecological Zones version 4 (GAEZ v4). URL: http://www.fao.org/gaez/.
