# Supplementary material for: Agricultural input shocks affect crop yields more in the high-yielding areas of the world
Source: Nat Food. 2023 Nov 9;4(12):1037–46. doi: 10.1038/s43016-023-00873-z (PMC10727984; doi:10.1038/s43016-023-00873-z)
Supplement: Supplementary file 2 — Reporting Summary [file 43016_2023_873_MOESM2_ESM.pdf]

nature portfolio

Corresponding author(s): Matti Kummu

Last updated by author(s): Sep 29, 2023

Reporting Summary

Nature Portfolio wishes to improve the reproducibility of the work that we publish. This form provides structure for consistency and transparency in reporting. For further information on Nature Portfolio policies, see our Editorial Policies and the Editorial Policy Checklist.

Statistics

For all statistical analyses, confirm that the following items are present in the figure legend, table legend, main text, or Methods section.

n/a	Confirmed	
☒	☐	The exact sample size (*n*) for each experimental group/condition, given as a discrete number and unit of measurement
☒	☐	A statement on whether measurements were taken from distinct samples or whether the same sample was measured repeatedly
☒	☐	The statistical test(s) used AND whether they are one- or two-sided<br>*Only common tests should be described solely by name; describe more complex techniques in the Methods section.*
☒	☐	A description of all covariates tested
☒	☐	A description of any assumptions or corrections, such as tests of normality and adjustment for multiple comparisons
☒	☐	A full description of the statistical parameters including central tendency (e.g. means) or other basic estimates (e.g. regression coefficient) AND variation (e.g. standard deviation) or associated estimates of uncertainty (e.g. confidence intervals)
☒	☐	For null hypothesis testing, the test statistic (e.g. *F*, *t*, *r*) with confidence intervals, effect sizes, degrees of freedom and *P* value noted<br>*Give P values as exact values whenever suitable.*
☒	☐	For Bayesian analysis, information on the choice of priors and Markov chain Monte Carlo settings
☒	☐	For hierarchical and complex designs, identification of the appropriate level for tests and full reporting of outcomes
☒	☐	Estimates of effect sizes (e.g. Cohen's *d*, Pearson's *r*), indicating how they were calculated

Our web collection on statistics for biologists contains articles on many of the points above.

Software and code

Policy information about availability of computer code

Data collection	no software was used
Data analysis	The analyses were conducted with the R programming language (version 4.2.0) using the RStudio integrated development environment (1.1.456). A custom random forest algorithm (Breiman, 2001) was used to analyze the data (randomForest 4.7-1.1). The analysis codes are available in GitHub https://github.com/ahvoa/shock_pub.

For manuscripts utilizing custom algorithms or software that are central to the research but not yet described in published literature, software must be made available to editors and reviewers. We strongly encourage code deposition in a community repository (e.g. GitHub). See the Nature Portfolio guidelines for submitting code & software for further information.

Data

Policy information about availability of data

All manuscripts must include a data availability statement. This statement should provide the following information, where applicable:

- Accession codes, unique identifiers, or web links for publicly available datasets
- A description of any restrictions on data availability
- For clinical datasets or third party data, please ensure that the statement adheres to our policy

All the input data are openly available from the sources mentioned in Methods. All the output raster files from the scenario results can be accessed in the data repository: https://doi.org/10.5281/zenodo.8381197. The analysis codes are available in GitHub: https://github.com/ahvoa/shock_pub.

Human research participants

Policy information about studies involving human research participants and Sex and Gender in Research.

Reporting on sex and gender	We did not have human research participants
Population characteristics	We did not have human research participants
Recruitment	We did not have human research participants
Ethics oversight	We did not have human research participants

Note that full information on the approval of the study protocol must also be provided in the manuscript.

Field-specific reporting

Please select the one below that is the best fit for your research. If you are not sure, read the appropriate sections before making your selection.

☐ Life sciences ☐ Behavioural & social sciences ☒ Ecological, evolutionary & environmental sciences

For a reference copy of the document with all sections, see nature.com/documents/nr-reporting-summary-flat.pdf

Ecological, evolutionary & environmental sciences study design

All studies must disclose on these points even when the disclosure is negative.

Study description	In this study, we modelled the effects of agricultural input shocks on global crop production. We utilized global gridded data on crop yields, fertilisers, machinery and pesticides using a random forest machine learning algorithm.
Research sample	Twelve globally important food crops were selected for analysis for which all necessary data was found: barley, cassava, groundnut, maize, millet, potato, rice, sorghum, soybean, sugar beet, sugarcane and wheat. The data collected for each crop include: yield, machinery, fertilizer application rate, pesticide application rate, share of harvested area under irrigation, as well as average precipitation and temperature of the investigated areas.
Sampling strategy	The availability of global gridded crop yield and agricultural input data limited the selection of food crops for the analysis. The resolution of the global data were 5 arc-minutes for all data, but the exact sample size varied across crops depending on the extent of their cultivation.
Data collection	Aino Ahvo collected the data from openly available online resources.
Timing and spatial scale	The data was downloaded during 2021-2023. All data utilized were of global extent.
Data exclusions	No data were excluded from the analysis.
Reproducibility	All the processing codes to reproduce the results are available at https://github.com/ahvoa/shock_pub
Randomization	When evaluating the quality of the model outputs, to minimise any effects of overfitting and spatial autocorrelation, the data in each climatological region were divided into 60 arc-min grids, which were randomly assigned to training and testing samples (75% and 25%, respectively).
Blinding	Blinding was not relevant because the study was built upon openly available data.

Did the study involve field work? ☐ Yes ☒ No

Reporting for specific materials, systems and methods

We require information from authors about some types of materials, experimental systems and methods used in many studies. Here, indicate whether each material, system or method listed is relevant to your study. If you are not sure if a list item applies to your research, read the appropriate section before selecting a response.

Materials & experimental systems

n/a	Involved in the study
☒	☐ Antibodies
☒	☐ Eukaryotic cell lines
☒	☐ Palaeontology and archaeology
☒	☐ Animals and other organisms
☒	☐ Clinical data
☒	☐ Dual use research of concern

Methods

n/a	Involved in the study
☒	☐ ChIP-seq
☒	☐ Flow cytometry
☒	☐ MRI-based neuroimaging
